# Toxicology as a nanoscience? – Disciplinary identities reconsidered

**DOI:** 10.1186/1743-8977-3-6

**Published:** 2006-04-28

**Authors:** Monika Kurath, Sabine Maasen

**Affiliations:** 1 Science Studies, University of Basel & Collegium Helveticum, ETH and University of Zurich, Schmelzbergstrasse 25, CH-8092 Zurich, Switzerland; 2 Science Studies, University of Basel, Missionsstrasse 21, CH-4003 Basel, Switzerland

## Abstract

Toxicology is about to establish itself as a leading scientific discipline in addressing potential health effects of materials on the nanosize level. Entering into a cutting-edge field, has an impact on identity-building processes within the involved academic fields. In our study, we analyzed the ways in which the entry into the field of nanosciences impacts on the formation of disciplinary identities. Using the methods of qualitative interviews with particle toxicologists in Germany, Holland, Switzerland and the USA, we could demonstrate that currently, toxicology finds itself in a transitional phase. The development of its disciplinary identity is not yet clear. Nearly all of our interview partners stressed the necessity of repositioning toxicology. However, they each suggested different approaches. While one part is already propagandizing the establishment of a new discipline – 'nanotoxicology'- others are more reserved and are demanding a clear separation of traditional and new research areas. In phases of disciplinary new-orientation, research communities do not act consistently. Rather, they establish diverse options. By expanding its disciplinary boundaries, participating in new research fields, while continuing its previous research, and only vaguely defining its topics, toxicology is feeling its way into the new fields without giving up its present self-conception. However, the toxicological research community is also discussing a new disciplinary identity. Within this, toxicology could develop from an auxiliary into a constitutive position, and take over a basic role in the cognitive, institutional and social framing of the nanosciences.

## Background

### Nanosciences shaping disciplinary identities

The nanosciences and -technologies are interdisciplinary research fields, which developed over the past two decades at the interface of physics, chemistry, biology, molecular biology and material sciences. The field researches the structure of material characteristics and functions on the nanometer scale. On the nano-scale quantum effects affect the behavior of matter in a consequential way. This phenomenon allows for the analysis of new material properties and a variety of applications. Nanosciences and -technologies are currently considered as leading innovation fields [1]. Hardly any other cutting-edge science has been able to generate such comprehensive expectations regarding its developmental potential. Globally, annual investment in research and development within this field exceed the billion-dollar mark [2]. Furthermore, almost no industrial nation can afford not to establish national programs and no academic institution, not to initiate research initiatives in this area. This opens the possibility for a number of scientific disciplines to participate in this area and adapt their research to related issues and objectives.

Apart from the scientific and economic euphoria, various social actors have started to debate the potential implications of these new scientific and technical developments. A variety of researchers and institutions have begun to investigate the potential ethical, social and environmental effects of nanosciences and -technologies. On the one hand, nano-scientists themselves have been addressing such issues, e.g., the joint founder of Sun, Bill Joy, who published a manifesto in the magazine Wired [3]. On the other hand, technology-critical civil society organizations focus their activities on nanosciences and -technologies, e.g. the 'Center for Responsible Nanotechnology' [4], the Action Group on Erosion, Technology and Concentration (ETC), Canada [5], as well as Greenpeace [6]. Furthermore, social and political institutions, like the Royal Society in UK [7], the European Commission [8] and several national technology assessment institutions [9] have addressed potential risks of nanosciences and technologies.

In addition, spectacular visions and Utopias as well as dystopic scenarios have been formulated and discussed in media and on the literary level. A well-known example is the 'grey goo' scenario, which, for example, was pointed out in the Drexler-Smalley debate and was addressed by Michael Crichton in his novel 'Prey'. The negotiation of fictions in the debate between Eric Drexler and the nobel laureate Richard Smalley focused on the question of the feasibility of molecular assemblers [10]. Drexler is seen as the founder of the idea of machines, which develop objects atom by atom and replicate themselves [11]. Smalley, as a representative of classical chemistry, tried to disprove the futuristic approach of Drexler. He argued that such Utopian ideas could encourage public fear of a loss of control over such machines and of their unlimited spread ('grey goo') and this in turn could damage the reputation of nanotechnologies as a whole. The taking up of grey goo' scenarios by the Prince of Wales, was widely discussed into the popular media and led to a Royal Society report on the chances and uncertainties of nano-sciences and technologies [12]. Relevant fictions have also appeared in novels of Anderson, Asimov, Bear and Stephenson [13]. Michael Crichton describes, in possibly his most famous novel, 'Prey', a 'grey goo' scenario involving the loss of control over nano-technologically manufactured micro robots, 'nanobots' [14].

Such discourses have crucially contributed to the demand for a moratorium on the technological development and production of nano-materials made by civil society organizations like for example the Canadian ETC-group [15]. Furthermore, a moratorium on the development of nano-materials was proposed to national government leaders at the world summit for sustainable development in Johannesburg 2002 [16]. In addition, the manifesto of Bill Joy is occasionally interpreted as a call for a moratorium stressing the unforeseeable risks of nanotechnologies. Joy makes comparison to the development of the atom bomb and pleads for deeper ethical reflection on the nanosciences and -technologies [3].

Beside these more future-oriented risk discourses, however, tangible health effects of particles at the nanometer scale have been detected by a variety of toxicological working groups [17]. So far, concrete findings regarding potential risks of nanosciences and -technologies focus on the health implications of particles at the nanometer scale. This field addresses those scientific disciplines, having methodological and textual experience in the investigation of the bio-reactivity of particles and materials for its analysis. In this context, toxicology as a scientific discipline plays a responsible role. Toxicology has traditionally examined the potential harmful effects of chemical or physical agents on biological systems. While nanosciences and -technologies do not yet form a coherent program, toxicology sees itself as contributing to the public discourse with technically clear and concise answers, broadly recognized in the media. Early exponents in the toxicological research community are already claiming the emergence of nanotoxicology as a new discipline [18] and a new journal with the title 'Nanotoxicology' has been launched in 2005 by the Taylor and Francis group [19].

Before explaining the aim of this study, our hypothesis and what will be reported in this article, we will give a short explanation of our theoretical model: The formation of disciplinary identities can be analyzed using theories of the development and differentiation of scientific disciplines [20]. Toxicology as a scientific discipline understands itself through its orientation toward externally defined problems like for example the supply of practical guidelines for the adjustment of toxic chemicals. Therefore, the concept of 'Finalisierung – finalization' [21] plays an important role in the analysis of the construction of disciplinary identities. The concept of 'finalization' focuses on the influence of internal and external factors and orientations within the development of science. Boehme et al. and van den Daele and Weingart developed this approach in the 1970s and were influenced by Luhmann's system theory and Kuhn's theses of scientific progress [22]. Van den Daele and Weingart base their theory on three variables influencing the differentiation of scientific disciplines: cognitive, institutional and political aspects. Cognitive aspects describe factors, which define science as an intellectual enterprise [20].

Furthermore, cognitive aspects specify the development of a discipline and consist of such things as internal structures and epistemic practices [23]. In contrast, institutional aspects focus on internal processes within scientific institutions, such as co-operation, communication and interpersonal relationships. Such processes determine science as a social operation system and they differentiate new research fields [24]. Political aspects are understood in terms of science-policy attitude and demand a 'product value orientation' from science. In this way, science is controlled by political interests and orientated toward the solution of specific, socially induced and politically defined problems like cancer- or environmental research [25]. Thus, in our study we will and use the term 'external aspects' instead of 'political aspects', considering beside of politically, also socially and economically relevant criteria. According to this account, disciplinary development takes place via different contexts: on the one hand, by cognitive and institutional aspects that are internal to science, on the other hand, by problem settings that are external to science [26]. However, we do not understand problem orientation in the sense of the direct intervention of society into the sciences. Rather, we consider problem orientation as external requirements that disciplines perceive and accept in a system-specific way [27].

For toxicology the following constellation results: On the one hand, problem orientation and context sensitivity, particularly with respect to (future) nano-technologies, contribute to the fact that toxicology has a substantial role to play in cognitive, institutional and social respects. The nanosciences could profit from institutionalizing toxicology, whose research directly meets the social requirement of security, as an already finalized partial discipline within the new interdisciplinary field. With the present close interconnection between the (nano-) sciences and society, this arrangement could allow toxicology to ascend from an auxiliary science into an increasingly constitutive position within the nanosciences [28]. In order to put into perspective the estimations and strategies of toxicology in this challenging state of transition, we revert to two further concepts: 'thought-style/Denkstil' and 'boundary work'.

In order to understand the development of scientific disciplines in light of the above mentioned aspects, theories that understand academic knowledge production as the collective achievement of a community of scientists in a particular research field will prove helpful. Such 'thought collectives/Denkkollektive' are representing assumptions and conditions, under which they are building up a certain knowledge, a prevailing doctrine and, in the terminology of Fleck, a 'thought style' [29]. According to Fleck, knowledge is never possible on its own, but only in the context of various presuppositions about it. Therefore, disciplines represent thought collectives whose style of thinking is shaped by their surrounding social, political and cultural context [30].

In periods of transition between thought styles, 'Denkstilwandel', the collective is not consolidated. No commonly shared views exist. In this phase no uniform thought styles can be identified; the collective shaping of identities is in flux. Cognitively and institutionally it is still unclear where the development will lead. Such considerations are also helpful for our study: In the phase of the 'nano-scientific challenge' we expected to find a variety of different positions and estimations of the present and future role of toxicology within or outside of the nanosciences.

Therefore, a third concept; that of 'boundary work' is helpful for describing the various possibilities that result from that assumption [31]. Thomas Gieryn has developed the concept of 'boundaries' to describe textual as well as institutional demarcations between 'science' and 'non-science ' [32]. In accordance with the concept of finalization (cognitive, institutional, social aspects, shaping the development of disciplines) mentioned above and the anticipated heterogeneity of the thought styles of scientists working inside a transforming field, the point of boundary work lies in the fact that the fixing of boundaries depends on contextual factors such as which the topics, questions and methods belong to 'our' field and which do not. Using the 'boundary work'-concept, we hope to show, in the case of toxicology, how scientific fields form their disciplinary identity by setting boundaries between new and old research fields.

Against this background, we will analyze toxicology as a scientific discipline, which is establishing itself within the field of nanosciences and -technologies. Herein, we are interested in what way toxicologists are producing their knowledge and how involved researchers assess their professional identity. Furthermore, we will analyze the cognitive, institutional and political aspects, shaping the disciplinary development. Herein, we will put a particular focus on the shifting thought styles of the particle toxicological community and their setting of boundaries with regard to the emerging fields of nanosciences and -technologies.

Hence, our hypothesis is that the disciplinary identity formation of toxicology, set against the background of its entrance into the nanosciences and -technologies, represents a still open-ended and rather incremental scientific development.

**Figure 1:**
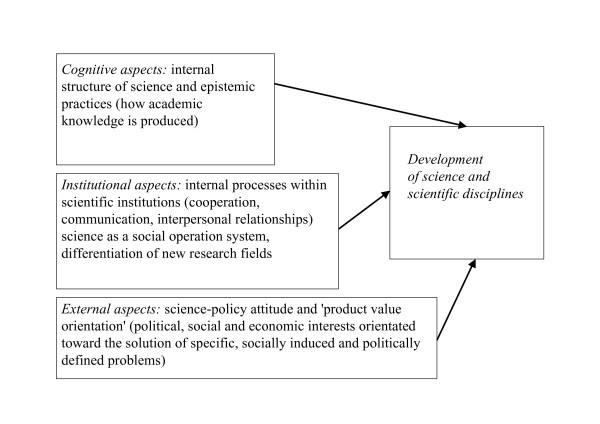
Finalization-concept: three aspects, influencing the development of science and scientific disciplines.

**Figure 2:**
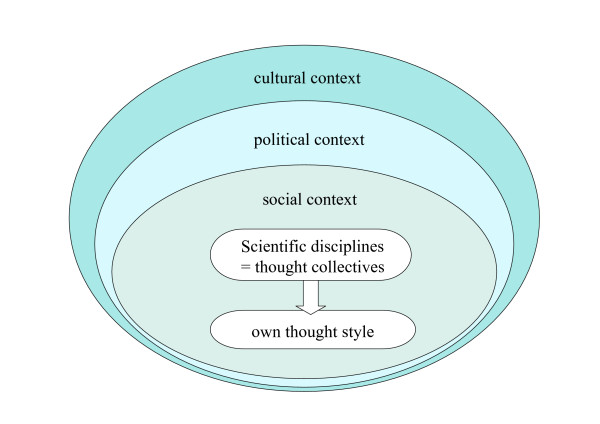
Concept of thought collectives and thought styles. Scientific disciplines represent thought collectives with a particular style of thinking, influenced by their cultural, political and social context.

## Methods

For the analysis of disciplinary identity-building processes, we selected a qualitative sociological research approach, based on the method of 'grounded theory' [33]. It consists of a case study analysis [34] within the research field of particle- and inhalation toxicology and an empirical study. We also worked with qualitative interviews and the problem-centered interview method [35]. This allows for the combination of narrative elements with a manual structure, which enables the consideration of background knowledge [36]. In our research we interviewed fifteen leading scientists from Germany, Holland, Switzerland and the USA, all of whom are analyzing the potential health impacts of nano-scale particles. We recruited our interview partners through the method of'theoretical sampling' [37]. With this method we did not chose a representative sample following the usual criteria of sample selection, e.g., the coincidence principle. We rather selected persons, participants, and representative institutions, with regard to their potential for contributing to the research project. Hence, neither the extent nor the characteristics of the sample were fixed in advance. In addition, theoretical sampling allows the selection of new participants, whose relevance only shows up during the research process. Sample selection was terminated when theoretical saturation was reached [38]. Theoretical saturation is reached when the insights gained per additional interview declines.

Qualitative research, and the approach of 'grounded theory' distinguish themselves in not working with preliminarily defined variables. Rather, the analytical codes are directly extracted from the interview-sequences. Along those codes, which build up the subsequent result section, the arguments are worked out [39].

## Results

### Knowledge-production and disciplinary identity formation in toxicology

Following a historic overview at knowledge-production in toxicology, we will present our views on disciplinary identity formation in toxicology through a discussion of citations from our interviews.

### Knowledge-production in toxicology

In this chapter, we will give a historical overview at the development of toxicology and its three-phase transformation into a scientific discipline. Furthermore, we will focus on the specific methods and practices with which toxicological knowledge-production enters the nano-sciences. Selected quotations from our interviews will be used to illustrate how toxicological identity formation is challenged by the cooccurrence of a previous, historically grown technical self-understanding (thought style) as well as the new requirements of participating in the nano-sciences.

Traditionally, toxicology examines the potentially harmful effects of chemical or physical agents for biological systems. It is also called the science of the poisons [40]. During its transformation into a scientific discipline, toxicology went through three phases: In the first phase, the health effects of selected substances were observed. First, toxicologists wrote down a phenomenology of poisons and remedies. The roots of such phenomenologies of poison can be retraced in the origins of the development of human medicine. In antique European, Arabic and Asian cultures, knowledge of toxic substances was inseparably linked with medical training and practice [40]. Furthermore, the science of toxin was closely linked to botany and the plant sciences in ancient Greece. Theophrastus, a pupil of Aristotle (372-287 BC) composed botanical works and gave detailed descriptions of medicinal and poisonous plants. His work has been designated as the beginning of modern botanies. Arab cultures developed chemical approaches in toxicology. In the Middle Ages, mainly southern European physicists like Maimonides (1135–1204) and Pietro de Abano (1250–1316) contributed to the identification of poisons [40].

A second phase covered the experimental approaches, used to examine the mechanisms of dose effect dependence. Paracelsus (1493–1541) is considered the founder of this phase. In the 16th century he developed a concept of poison, which is still applicable today. He thereby initiated a turn away from a merely descriptive analysis of phenomena and the categorizations and listing of poisons then found in experimental and analytic research approaches. Furthermore, he began to use his knowledge for therapeutic applications, that is, he used 'poisons' for beneficial effects. The knowledge gained in a variety of scientific and medical disciplines supported the transformation of toxicology in the 18th and 19th centuries from knowledge-production based on experience into a concept-based scientific field [41]. In the early 20th century, toxicology was established as a natural scientific discipline clearly demarcated from pharmacology, medicine, chemistry and biology [40]. In a third phase, toxicology increased in relevance through its emergence as a testing discipline and attendant research science. This happened in the second half of the 20^th ^century in the context of intensive scientific and technological growth, accompanied by hazardous incidents and social controversies. Through the development of guiding principles for the regulation of toxic chemicals, work place safety and public health, toxicological knowledge became useful for politics and policy-making in various industrial nations. With its knowledge of the health and environmental effects of new materials and substances, based on quantitative laboratory studies, toxicology developed a comprehensive network for the measurement and categorization of the dose-effect-dependence of the most prominent industrially used materials. The investigation of the toxicity of particles is closely linked historically to mineral fibers like asbestos and industrial activities, like coal mining. Thus, the European community for steel and coal (ECSC) contributed to the establishment of the research field of particle toxicology [42]. By developing concrete, measurable testimony about risk and safety, toxicology established itself as an attendant, testing discipline and monitorial authority. They are cover the identification, quantification and prevention of the unfavorable side-effects of chemicals. In addition, safety regulations for job descriptions, and tolerance limits for chemical additives in food and water were determined [43]. Hence, as one interview partner holds, toxicology became dependent on industry and politics. Toxicology needs research funds from industry and politics, which in turn need applicable toxicological knowledge.

"For politics and industry our research is absolutely relevant and necessary. Therefore we need their support." (Toxicologist II, German research center)

Knowledge-production within toxicology mainly focuses on quantitative in-vitro and in-vivo studies [44]. In-vitro studies cover laboratory studies of cell lines which are specially bred under variable conditions. In-vivo studies focus on the reaction of the organism as a whole to the admission of and exposure to certain substances. They mainly involve animal tests with conventionally bred or transgenic rodents in the laboratory and, to a more modest extent, clinical trials with humans, as a researcher at a Swiss university, working in the field of inhalation toxicology, holds:

"*Our possibilities to conduct studies with humans are extremely limited. Therefore, we try to develop models. In-vivo, I have been working with small rodents, like rats and hamsters. Recently, I have also started to work with mice. In particular, I use transgenic mice, which I consider a very good model for specific questions. With the second model we are trying to reconstruct respiratory epithelia in- vitro, in order to observe particle-lung interactions." (Biologist II, Swiss university)*

Toxicology represents an interdisciplinary research field. It uses up-to-date methods and techniques, which overlap with neighboring disciplines like chemistry, pharmacy and medicine. Since the 1990s it has also become involved in the field of molecular biology. At the same time, it underwent a paradigm-shift in its research practice from high to deep dosages [45]. In the opinion of several toxicologists, particularly in Europe, toxicology's interdisciplinary character is a crucial precondition for doing research on particles at the nano-scale level:

"We work together with the material sciences, with chemists, physicists and engineers. Furthermore, the topic is connected with meteorology and thermodynamics. In toxicology, the spectrum is even broader. The classical toxicologist is usually a biologist, biochemist, bio-physicist, pharmacist or bio-engineer. And epidemiology goes together with pure mathematics, particularly in the case of statisticians and epidemiologists, who have a medical or a scientific background, for example physics or chemistry, but primarily medicine." (Toxicologist I, German research center)

## Discussion

Toxicology thus established itself in the late twentieth century as a classicalexamination discipline and testing science in the course of efforts of various industrial nations to regulate toxic chemicals. Research questions within this field focus on current social and political issues. The adaptation of its research agenda to external problem definitions enables toxicology to focus on a problem- and application-oriented form of knowledge-production, which incorporate the latest approaches and practices. Relevant scientific investigations of the bio-reactivity of industrially manufactured particles on the nano-scale level, and concrete statements about their health implications, have enabled toxicology to establish itself as a leading discipline. The forms and functions of toxicological knowledge production are relevant in a time when society demands consideration of its own requirements. Toxicological research promotes ends that are not only profitable, but also result in safe products and procedures. Here, the subdisciplines of particle- and inhalation toxicology in particular play a central role [42]. The research field of particle toxicology, as a subdiscipline of toxicology, developed in the context of the study of lung disease, particularly as it occurs in the mining industry [42]. The central organ of a particle or inhalation toxicological approach is the lung. Furthermore, mainly insoluble materials are analyzed and scientists often work with traditional toxicological methods and approaches [42]. In the course of intensifying concern over air pollution, exhaust fumes and smog, the research field of particle toxicology gained in importance.

As a problem-oriented science, toxicology gets the chance to move in the periphery of problem-oriented but still not yet sufficiently formed cutting-edge research in the nanosciences and to profit from research funds within these fields. Nevertheless, in societal discourse, in the media and in politics, toxicologists are considered the experts with regard to the relevant questions. In our entire research area we found only two research groups, focusing on the health risks of particles in the nanometer scale, which did not characterize themselves as toxicologists. Nevertheless, these groups are methodologically, textually and technically working closely together with toxicology and in close exchange with toxicological working groups. They distinguish themselves from toxicology, arguing that they, unlike toxicology, examine entrance mechanisms by which particles enter into the body and do not focus on material effects within the organism. Furthermore, they claim to work with deeper and more realistic dosages and a minimum number of laboratory animals. Nevertheless, they produce knowledge comparable to that produced by toxicological working groups. Press coverage on the health risks of particles in the nanometer scale mainly focuses on toxicologists as the experts in this field [46].

### Shaping disciplinary identities in toxicology: cognitive, institutional and external aspects

In our study, we found three aspects shaping the disciplinary identity of toxicology against the background of its entrance into the research field of the nanosciences and -technologies. First, science-internal factors like cognitive and institutional aspects play an important role. In our study, we understand cognitive aspects as research objects, approaches, research- and everyday-practices and resultant findings. Institutional aspects cover scientific contexts, like access to research funds and the reputation of a discipline. Under external aspects (orientation to social problems) we subsume negotiation processes in matters of definition and orientations to therapeutic approaches. We will group our results around these three aspects, and include quotations from our interviews.

### Cognitive aspects: risk research as tradition and transition

The health effects of particles on the nanometer scale, discussed in science, politics, the media and by the public, are comparable to the risk concepts of toxic chemicals. Through the analysis of the toxicity of chemical substances, comparable approaches are selected and similar insights are achieved. When compared to a variety of materials, pollutants and substances being deposited into the environment, the discussed health effects of nano-scale material display their chronic toxicity. This, and its longtime research experience with particles on the micrometer and ultrafine level has enabled toxicology to establish itself as a scientific discipline in the risk analysis of particles on the nanometer scale [47]. The analysis of the health implications of ultrafine particles in laboratory trials took place with well-defined, specially produced reference particles. The use of reference particles enabled the comparison and reproduction of particular test arrangements. Such reference particles are similar to today's industrially manufactured nanoparticles. An interview partner, who has been working for over thirty years in this field, stressed that toxicological groups already worked with nano-particles before the term 'nano' was established.

"For us, the term 'nano' is old hat, we have always been 'nano' now for more than 10 years, although we did not use the term 'nano'." (Toxicologist I, German research center)

Toxicology established itself in the analyses of health implications of micrometer particles in the last thirty years. A close methodological and textual connection exists between research on unintentionally and combustion-produced *ultrafine *environmental particles and well-defined selectively produced particles on the nanometer scale. Particularly, experience from the analysis of the health impacts of particles on the micrometer scale is seen by several interview partners as a central precondition for doing research with particles on the nanometer scale, as another toxicologist from a German research center holds:

*"I started with micrometer particles. 98% of my knowledge and experience is based on micrometer particles. [...] thirty years ago, I was concerned with particles, sized between 0.5 and 5 micrometers. And then the ultrafine particles came up. That was ten years ago and since then I have been doing something similar with the ultrafines*." (*Toxicologist III, German research center*)

Besides work with particles on the micrometer scale, constitutive research on ultrafine particles supplied important insights and preconditions for working with selectively produced particles on the nanometer scale. Based on their experience with the bio-interactions of those particles, several interviewpartners are disclosing the behavior of selectively produced nano-particles.

*"The industrially manufactured nano-particles are materially comparable to environmental particles. Therefore, research within these fields can well be combined. [...] Our experience with ultrafine particles is of high importance for analyzing the risks of nano-particles. Along with ultrafine particles, we began to use nano-test particles to investigate certain mechanisms." *(*Toxicologist II, German research center)*

The transition from the 'ultrafine-' to the 'nano' scale often happens inconspicuously. Alongside research with ultrafine particles, similar experiments are repeated with selectively produced nano-particles. As a rule, however, particularly the German research groups, we analyzed, sought a clean distinction between established and new research fields.

*"I don't want to give the impression that we are only working with ultrafine dust, with combustion stuff. We also work with the typical synthetic nano-particles [...] thus, we do both: We work both: on the combustion side with fly ash and environmentally relevant particles as well as with nano-technological particles. We clearly divide this into two different projects, with the appropriate nomenclatures. "*(*Toxicologist IV, German research center)*

Most insights into the potential health effects of particles on the nano-scale level have resulted from toxicological approaches, on methods, and were achieved in toxicological research groups and laboratories. The most frequently observed health effects and bioreactive phenomena of materials on the nanometer scale are inflammatory cell reactions resulting from the deposition of such materials in the lung [48]. The transport of nano-scale particles through the lung into the blood was also observed [49]. In addition, the particles were found deposited in the body and to have even overcome the blood-brain barrier [50]. In fact, nano-scale particles were found in all organs of the body [51]. This fact was also reported by a researcher in the field of inhalation toxicology at a Swiss University:

"We found the particles distributed in the entire lung four hours after their admission. Furthermore, we found them in the blood, from where they can easily be distributed into the whole organism [...] a colleague of mine at the GSF in Munich found particles in the liver, in the kidney, in the heart and even in the brain. Epidemiological studies have shown that with accumulating concentrations of fine dust in the air, certain diseases, such as cancer, and heart, circulation and respiratory problems, are increasing." (Biologist I, Swiss university)

Furthermore, some results show that nano-scale particles are transported into the brain by olifactory nerve cells [52] and are involved in neurodegenerative changes [53]. The same researcher continues:

*"Nano-particles are transported along the olfactory nerves into the brain. We do not know what happens there. Besides that, there is a study showing that the histological picture of Alzheimer patients, who died at the age of 70 or 80 years, closely match a picture of the brain of accidentally killed 30 year old persons who lived in areas with a strong particle-load. *"(*Biologist I, Swiss, University*)

A research group at *the GFS – Research Center for Environment and Health *(in the Helmholtz community) in Neuherberg near Munich developed a technique for quantifying the health implications of particles within the nanometer scale. It conceives of risk and a shortening of lifetime and expresses this in the number of lost life years [54]. Thus, the risk is constructed through the 'lifetime-shortage' factor as a measurable, conceivable and concrete quantity. A researcher of this group argues:

*"According to our epidemiological studies, long-term exposure with high concentrations of environmental aerosols can shorten a life span by one year. In Germany, between 4'000 and 10'000 persons per year are affected." *(*Toxicologist I, German research center)*

## Discussion

In summary, we argue that, in this early phase of identity formation, toxicology strongly relies on current methods, practices and approaches. In parallel with its well-established analysis of the health effects of particles on the nanometer scale, toxicology is building for itself a new research field through its analysis of the potential impacts of nano-scale particles. Entering into this field, toxicology brings with it basic knowledge from the analysis of micrometer and ultrafine particles. In this way, the transition to a new field proceeds incrementally; this careful movement is particularly well-articulated in the quotation: "we have always been nano". This is as it were, the secured position from out of which nano-specific questions and procedures can be necessarily conceded. In the next section we will address the question of how – alongside the cognitive aspects -institutional conditions, like funding acquisition strategies and the reputation of toxicology as a science, are also shaping disciplinary identities in this research field.

### Institutional aspects: acquisition and reputation

Besides the cognitive conditions, discussed in the previous section, institutional aspects are also shaping the formation of the disciplinary identities of toxicology in the context of its entrance into the research field of nanosciences and -technologies. The increasing scientific, political and economic interest in nanosciences and -technologies have opened considerable funding sources for this field. Thus, research projects for the investigation of the health implications of industrially manufactured nano-particles are usually more generously supported than similar investigations of particles originating from combustion processes. For this reason there is an interest in expanding the research breath of the nano-sciences, as a toxicologists of a German research organization holds:

*"If you write the term 'nano' into your research grant applications, the probability to get funding is much higher than if you use the term *'*ultrafine'*."*[...]*(*toxicologist, German University*)*" 'Nano': this is a fashion and naturally also a proposal strategy. If I applied for research funding on ultrafine dust at the European Union, that would be old hat. It was already done in the 1970s and the 1980s. However, if I applied for funding for a project on the influence of nano-particles, then everything looks quite different." [...] (Toxicologist II, German research center) " Furthermore, it was a question of funding. In the European Union, research on ultrafine particles was promoted less after 'nano' emerged and, since then, we have also been trying to get funding for 'nano'." (Toxicologist I, German research center)*

Besides funding strategies, toxicology's traditional historical role as a testing science enabled it to also enter into the new research field. The increasing growth of research and development within the nanosciences and -technologies made risk assessments inevitable. Hence, different material research institutes established research groups in toxicology as an accompanying testing science. Furthermore, they involved toxicology within their own field. As a German toxicologist argues, toxicologists were expected to analyze the potential health implications of materials on the nanometer scale, which are researched and developed within these institutes.

*"Our research institute has laboratories for nano-technology, material research and chemistry that are working with nano-materials. Our task is to accompany those technological developments with toxicological research*." *(Toxicologist II, German research center)*

In its role as an attendant testing science, several toxicologists, particularly in Germany but also in Switzerland complained of toxicology's lack of prestige. Since their research aims at discovering potential hazards, they are only able to publish their data if they have found a health effect for a substance or a product. As this is socially perceived as unfavourable, they feel themselves to be the bearers of bad news. In our interviews, we detected a certain disillusionment. In particular, toxicologists seem to lack the possibility of publishing socially favourable results such as that for a certain substance or product no danger could be proven, in renowned high-impact journals. Furthermore, some toxicologists expressed discontent with their financial situation and their lack of scientific and political appreciation. Hence, among toxicologists, particularly in the German research community the view dominates that productive disciplines enjoy a higher reputation and can publish their research results more easily and in higher-rated journals, as German toxicologists argues:

"If you work in toxicology, you only have negative results." [...](Toxicologist II, German research center) "My highest goal would be to get a safety study published in Nature. That is the problem, we can only publish negative effects. When we find positive, or rather no, effects, we cannot publish them. When we discuss that a particular substance is not toxic, this is fine for society but bad for us as scientists, since we measure research quality based on output. If a geneticist finds a new gene, a new gene product or a regulator in his laboratory, then he has a new paper. This is very simple. And the paper, if this is a basically important process, will be accepted in Science, Nature or somewhere else. This is not possible in our field. If we tested ten substances and they all turn out to be harmless, then we have just spent five years without publishing anything. As a toxicologist you hardly ever want to be in Nature or Science. Being there, you must have found a substance so toxic that you would prefer it did not exist. That is the difficulty with toxicology. But nobody recognizes that. The investors do not realize that, and neither do our clients nor society. Everybody says: Toxicology is expensive and brings nothing – from an economic perspective." (Toxicologist III, German research center)

Like ethics, toxicology assists neighboring disciplines in an accompanying and advisory way. It is basically a testing science. By turning its attention to risk, it often sees itself as being ill-reputed as a brakesman, a spoilsport, a critic or an unloved child. For toxicology, this role is unappealing. According to some interviewed German toxicologists, productive disciplines seem to undervalue the constructive aspect of toxicological knowledge production, as another German toxicologist argues:

*"Toxicology is usually seen as a brakesman and spoilsport. Toxicology focuses on implications that an engineer does not necessarily see, but which could be of great importance for him. This means that there absolutely is a knowledge gain resulting from the analysis of hazardous implications. Toxicology should be a constructive element with the development of nano-technologies. Collaborations between developers and those who focus on implications are crucial. If this happens at as early a stage as possible, then this is – according to my perception – no handicap, but rather a moment of creative research, positively stimulating the whole process*." *(Toxicologist I, German research center)*

## Discussion

Institutional aspects like finding acquisition strategies and toxicology's traditional role as a testing science enable its entrance into the research field of nanosciences and -technologies. Moreover, the toxicological research community expresses the desire for higher outside appreciation. Toxicology aims at constructively accompanying and creatively influencing controversial cutting edge research. Furthermore, toxicology prefers the role of the productive partner rather than the brakesman. This aim represents kind of a crucial test – at least in terms of the transitional phases for evolving disciplinary identities. While the braking and warning function belongs to its classical scope, toxicology nevertheless fears that it will not even be able to fulfil those functions. Thus the question occurs, whether these functions detract from ('spoil sport') or support toxicology's disciplinary reorientation, since it now sufficiently fulfils social requirements for safety. We will focus on this still undecided question under the title of problem-orientation in the next section. In doing so, we will also analyze the impact of toxicological participation in the therapy-oriented research of the nanosciences.

### External aspects: problem orientation and negotiation processes

Besides those aspects internal to science, orientation to science-external research questions and problems also shapes the disciplinary identity of toxicology. One important example is the orientation of toxicological knowledge-production toward nanoscientific approaches to therapeutic issues in pharmacology. The aim of this research area is the development of mobile drug carrier systems based on nano-particles. Here, nano-particles are used as transport systems for therapeutic agencies. Due to its specific characteristics, a medication can be directly applied to the effected location in the body [55]. Toxicology can adapt its insights into specific particulate and material properties and structures, developed in in-vitro and in-vivo studies, to the production of bio-inert particles with minimal health effects. Besides its function as a testing discipline, toxicology has the opportunity to adapt is knowledge to the health implications of particles on the nanometer scale range to the production of particles, for therapeutic use in pharmacology, which do not verifiably harm biological systems. We found this argument in the European as well as in the US-context.

" *'Nano' offers an enormous potential for toxicology. For example, we are able to develop bio-inert particles." [...] (Toxicologist, US-university) "A positive approach is therapy. This is, at the moment, a good idea, and one for which we have filed a project application. We will not examine any particular therapeutic aspects, but will rather aim at finding out how a nanoparticle should be designed, and what surface properties it must have in order to not cause any reaction in the organism. If I created such a particle, I could load it with a medicament or equip it with receptors such that these would then be carried into the cells. The real positive thing about this approach is that you can in the end, use it therapeutically, for example, in tumor therapy. You can attach to these cells through certain receptors, which the tumor is expriming. It will be absorbed into the cell and the agent will then be developed specifically in the tumor cells. In contrast, simplifying things, conventional chemotherapeutic agents are simply given to the organism and the tumor cells are more intensely damaged than the normal cells, as they have a higher turn-over. I would see this as a positive aspect of toxicological research." (Toxicologist IV, German research center)*

Besides the therapeutic orientation, definitorial demarcations seem to be another externally induced influence on disciplinary identity shaping in toxicology. In particular, the toxicological research community does not share a uniform attitude regarding the question of when a particle can be called a 'nano-particle'. While one part of the community argues for a merely size-oriented definition, others demand an origin-oriented distinction. Through this controversy, discussions and negotiation processes are developing concerning the demarcation of the research field of the nanosciences and -technologies. Part of the toxicological research community is using a purely size-oriented approach and is including all particles within the nanometer range (with at least one dimension smaller than 100 nanometers or 10^-7^-10^-9^m) under the term 'nanoparticle', as toxicologists in Germany and in the Netherlands argued.

*"Nano particles are particles which are at least in one dimension smaller than 100 nanometers. Through this, also nano tubes and plane particles are also incorporated. With the ultrafine, all three dimensions must be smaller than 100 nanometers. Therefore, we are classically ultrafine and to that extent also nano. Because ultrafine is always nano as well*." *(Toxicologist I, German research center)*

This enables the researchers involved to call their research on ultrafine particles 'nanoscience', and to partake of the rich funding opportunities in this field (see also section 4.2).

Toxicologists who plead for an origin-oriented distinction, argue that randomly emerging particles on the nanometer scale, usually resulting from combustion processes, should be called 'ultrafine-' or 'ambient air particles'. Furthermore, such particles usually consist of complex material mixtures. Only industrially manufactured, materially clearly defined particles within this size-range ought to be called 'nano particles'. We found this argument as well in the European than in the US context.

"*Concerning nomenclature at the very least the basic need for a distinction between clearly defined, engineered nanosized particles and those accidentally released into the environment, should be met [...] Defining complex material mixtures, I'd rather use the general term 'ultrafine particles'. For engineered materials, I would use the term nano-material. The term 'nano-material' implies technical design and intentional manufacture. The size range of particular ultrafine particles only coincidentally lies in the nanometer scale. Therefore, I would use terms like 'combustion particle' or 'environmentally relevant particle' for particles unintentionally released into the environment, and definitely not the term 'nanoparticle'." (Toxicologist II, German research center)*

As a compromise between the two positions, other members of the toxicological research community suggest the use of the term 'nano-scale particle' for both kinds of particles. This still size-oriented concept permits research in the field of ultrafine particles to be included under the term 'nano', and it allows such research to profit from the considerable research funds available to the nanosciences. At the same time, this definition is seen as allowing the desired demarcation for public-risk discourse.

*"Therefore, I'd suggest the term 'nano-scale particle' as a comprehensive definition for environmental particles within the nanometer scale. *"(*Toxicologist, US-university*)

## Discussion

The openness of the term 'nano', and the vague boundaries of the nanosciences and -technologies leave considerable space for toxicology to form its own disciplinary identity. Research fields, like the analysis of the health risks of ultrafine particles, can be subsumed under the 'nano-sciences'. The definitions used so far are set less for scientific than for political reasons, such as funding acquisition strategies and carreer interests. Hence, it is feared that a merely size-oriented definition could transfer public concerns over the health effects of ultrafine particles onto the entire area of the nano-sciences. This would, according to some, provoke public resistance to the nanosciences as such. Therefore, there is an interest in narrowing the research field of the nanosciences. Yet such demarcations also decide, whether another, quite relevant discipline (here: Toxicology) should obtain access to what is distinguished as a key technological field, and so also to the considerable research funds available to that field. In the negative case, toxicology would remain a less spectacular side-field with more modest funds. Therefore, the term 'nano' is not only rewriting a research field, with its specific questions and methods, but it also acts as a funding acquisition strategy and a reputation boost for individual researchers and groups. The entry of toxicology into the nano-sciences is enabled not only by its long research tradition in the analysis of the health implications of environmental and combustion particles on the micro- and nanometer scale, but as much by its taking advantage of an ambiguity by definition and vague demarcations of the research field of nanosciences and -technologies

Besides the social negotiation of definitioned demarcations, orientation to social issues also plays a substantial role in shaping disciplinary identities in toxicology. By participating in the development of therapeutics, like medical transmitter systems, toxicology sees a chance to leave behind its cognitively less attractive status as a purely evaluative field, and to develop from a classical testing science into a productive discipline. The entrance into the research field of the nanosciences and -technologies is seen as an opportunity for toxicology to transform its disciplinary identity and its mode of knowledge production. Whether such a transformation is actually taking place, and is introducing a new disciplinary identity, and whether toxicology will establish itself as a productive nanoscience, will be discussed in the final section.

## Conclusion

### Toxicology as a nanoscience?

The establishment of socially highly esteemed research fields opens up possibilities for a variety of scientific disciplines to participate within innovative research questions and partake of available research funds. Controversial issues like the potential risks of nanoscientific research, open the field to disciplines like toxicology that are qualified in the analysis of such issues, due to their cognitive and institutional background. By entering the emerging field of nanosciences and -technologies, toxicology is not only faced with the question of its contribution to current social debates on potential risks, but furthermore – and this is the central theme of our article – also with implications for its own disciplinary identity. Is toxicology or will it become, one of the constitutive nanosciences and -technologies?

Within the field of the nanosciences and -technologies, toxicology is contributing significantly to risk research through its epistemic and ontological tradition as a testing science. In this, its orientation toward externally given problem definitions, such as the supply of concrete statements on the health implications of particles within the nanometer scale as a basis for potential regulation, plays an important role. Problem orientation and context sensitivity, particularly toward (future) nano-technologies, enables toxicology to undertake an identity-shaping role, from a cognitive, institutional and social perspective. However, in this phase of the 'nano-scientific challenge', different positions and perceptions exist on the current and future role of toxicology within or outside of nanosciences. Hence, the formation of toxicology's disciplinary identity is shaped by cognitive aspects, such as its long research experience on the health effects of different materials, and its production of practical knowledge bases. Furthermore, institutional aspects, such as funding acquisition strategies and questions of reputation are shaping its disciplinary identity. Currently, toxicology finds itself in a transitional phase. The development of its disciplinary identity is incremental, open-ended and underdetermined.

All of our interview partners stressed the necessity of repositioning toxicology. However, they all suggested most different approaches. While some exponents of particle toxicology are already propagandizing the establishment of a new discipline -'nanotoxicology'- others are more reserved and demand a clear separation between traditional and new research areas. Furthermore, some toxicologists are afraid of encouraging social controversies over the potential risks of nanotechnologies. Hence, they aim to demarcate the field of nanosciences and -technologies on the basis of terminology. However, this competes with the interests of neighboring disciplines, also interested in benefiting from the research funds available within this field.

In both cases, aspects of 'boundary work' can be observed. By expanding its boundaries, participating in new research fields, continuing its traditional research, and only vaguely defining its research area, toxicology is trying to find its way into the new field without giving up its current disciplinary self-conception. Hence, there are a variety of possibilities: Alongside a general technical reorientation (change in thought-style), it is equally possible, if not even more probable, that toxicology is responding with external and internal differentiation. Thus, it faces current challenges (nanotoxicology) without neglecting its traditional expertise (toxicology as a testing science). Why choose when you can have both?

The development of a discipline into a new research field is shaped by science-internal aspects (cognitive and institutional) as well as by an orientation toward external problem definitions. In the transitional phases of disciplinary development, research communities do not act consistently. Rather, they establish diverse options by canceling definitional, epistemic and ontological boundaries. The fact that toxicological knowledge of the bio-reactivity and material behavior of a variety of substances and materials can be put to therapeutic uses, plays a substantial role. In particular, this therapeutic orientation enables toxicology to transform from its traditional role as an attendant and testing science into a 'productive' discipline. In this way, toxicology can 'ascend' from being an auxiliary science into a tendentious constitutive position. The question of whether context and the disciplinary requirements of certain problems not only enable the establishment of a discipline within an influential research field, but, was assumed in the case of the therapeutic orientation of toxicology in the nano-sciences, also lead to a transformation within the previous discipline cannot be answered completely. The positions currently vary. This is also the assessment of Vicky Colvin, who sees the role of toxicology in the nanosciences as one between "gatekeeper" and "player". According to her: "The paradigm shift really is not seeing toxicology as a gatekeeper but seeing toxicology as a point of information that allows you to generate more biocompatible materials" [56].

Nevertheless, we assume that also here the shaping of disciplinary identities will proceed incrementally and opportunistically. This will not only happen through a comprehensive new formation of toxicology in the sense of a paradigm-shift (nanotoxicology) and not only through the expansion of its classical scope (toxicology as a testing science also within the field of the nanosciences and -technologies). Rather, this will happen through internal differentiation i.e., both of the above as well as external differentiation. Hence, it is possible that individual researchers or research groups working in toxicology will migrate into neighboring disciplines like 'pharmacy' or 'nano-medicine'. Everything still seems possible, and everything at the same time: Why choose when you can have everything?

## Competing interests

The author(s) declare that they have no competing interests.

## Authors' contributions

Both authors made substantive intellectual contributions to this article. MK carried out the interview survey and wrote the text. SM participated in the design of the study, the evaluation, and developed the theoretical framework.
